# Eight rare urinary disorders in a patient with Kallmann syndrome: A case report

**DOI:** 10.1097/MD.0000000000022936

**Published:** 2020-10-23

**Authors:** Huining Tian, Zi Yan, You Lv, Lin Sun, Xiaokun Gang, Guixia Wang

**Affiliations:** Department of Endocrinology and Metabolism, The First Hospital of Jilin University, Changchun, China.

**Keywords:** Kallmann syndrome, renal agenesis/dysgenesis, urinary disorders, *KAL1*

## Abstract

**Rationale:**

Kallmann syndrome (KS) is a rare inherited genetic disorder characterized by hypogonadotropic hypogonadism and hyposmia/anosmia. Early diagnosis is the key to timely treatment and improvement of prognosis in patients with KS. As the most common complication of KS, renal agenesis can provide clues to early diagnosis and treatment for KS. In this article, we report a case of KS with 8 rare urinary disorders for the first time.

**Patient concerns:**

A 19-year-old Chinese man presented with 8 rare urinary disorders and a history of bilateral cryptorchidism came to us for micropenis, hyposmia, and delayed puberty.

**Diagnosis:**

The patient presented with hyposmia, low levels of sex hormones and showed a weak response to the GnRH stimulation test leading to a diagnosis of KS. Two missense mutations were found in further whole-exome sequencing: 1) Kallmann syndrome 1 (*KAL1*) gene in exon11, c.1600G > A, p. Val534Ile; 2) Prokineticin receptor 2 (*PROKR2*) gene in exon 2, c.533G > A, p. Trp178Ser. which led to a diagnosis of KS.

**Interventions:**

The patient underwent replacement therapy of human chorionic gonadotropin (HCG) and human menopausal gonadotropin (HMG). The patient had previously undergone six surgeries for cryptorchidism and urinary disorders.

**Outcomes:**

The patient's puberty retardation was effectively alleviated. His serum testosterone (T) reached a normal level (8.280 nmol/mL). During the follow-up period, he presented with Tanner stage II pubic hair development.

**Conclusion:**

In this article, we report 8 rare urinary disorders with missense mutations of *KAL1* and *PROKR2* in a case of KS. Among them, bilateral giant kidneys, urinary extravasation of right renal, bilateral megalo-ureters, left ureteral terminal obstruction, bilateral renal cyst and bladder emptying disorder are reported for the first time, which enrich the integrity of urinary disorder types and provide clues to genetic counseling in patients with KS.

## 1 Introduction

Kallmann syndrome (KS) is a rare inherited genetic disorder characterized by hypogonadotropic hypogonadism and hyposmia/anosmia.^[1]^ Studies have suggested that KS is associated with more than 20 genes and the mutations in these genes account for approximately 30% of all KS cases. These genes appear to involve in the migration of a group of neurons specialized in the sense of smell as well as neurons producing gonadotropin-releasing hormone (GnRH). KS may present as either a sporadic or a familial case, which contains 3 modes of inheritance: autosomal dominant, autosomal recessive, and X-linked recessive inheritance.^[2]^ The relationship between clinical phenotypes and gene mutations may provide clues for early diagnosis and therapy. Renal agenesis is considered as a signature phenotype of *KAL1* mutations and can be used as an early marker for genetic screening.^[3]^ In this article, we for the first time reported a diagnosis of KS in a 19-year-old young man with 8 rare urinary disorders demonstrating a conservative change in the *KAL1* gene (c.1600G > A, p. Val534Ile) and the *PROKR2* gene (c.533G > A, p. Trp178Ser) in this article.

## 2 Case presentation

**Figure 1:**
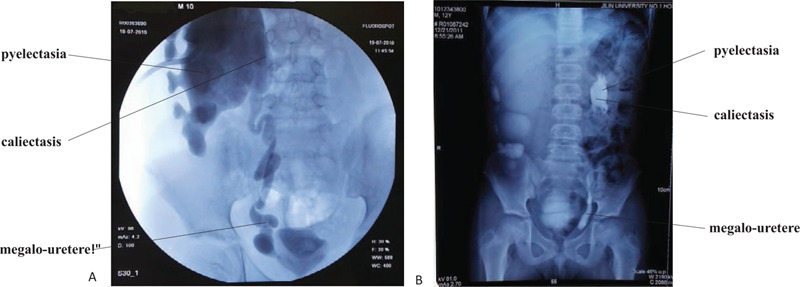
Pyelography results. A. Shot at 15 minutes after pyelography: right pyelectasia, right caliectasis, right uneven megalo-ureter. B. Shot at 15 minutes after pyelography: left pyelectasia, left caliectasis, left uneven megalo-ureter.

**Figure 2:**
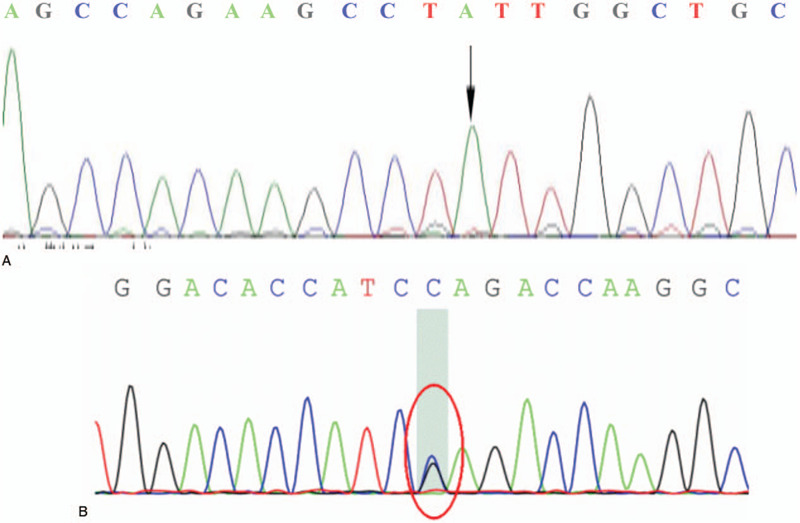
Whole-exome sequencing. A. Missense mutation of KAL1 gene in exon11, c.1600G > A, p. Val534Ile. B. Missense mutation of PROKR2 gene in exon 2, c.533G > A, p. Trp178Ser.

The patient is a 19-year-old Chinese male who presented to our hospital in 2015 for delayed puberty and experienced hyposmia for 3 years. The patient declared that he had experienced hyposmia for 3 years, but he ignored the symptom due to a history of nasitis. There is no relevant history in his family. In 2005, he accepted bilateral testicular traction and fixation for bilateral cryptorchidism, and received HCG (doses unknown) injection after the operation. Then, he was diagnosed with a complex of urinary dysfunctions, including bilateral giant kidneys and hydronephrosis, traumatic rupture of right renal, urinary extravasation of right renal, bilateral megalo-ureters, left ureteral terminal obstruction, bilateral renal cyst, and bladder emptying disorder. The patient underwent 5 operations for some of the above urinary disorders (Table 1). Pyelography results prior to a surgical operation were shown in Figure 1. He was 167 cm tall and 62 kg in weight (body mass index [BMI] = 22.3 kg/m^2^). At the age of 15, no pubertal development (no pubic hair) was noted. His penis was 2 cm in length and testicles were both 1.5 ml in volume. He had decreased levels of serum follicle-stimulating hormone (FSH 0.855 mIU/ml, normal range 1.27-19.26 mIU/ml), luteinizing hormone (LH 0.385 mIU/ml, normal range 1.24-8.62 mIU/ml), and T (0.720 nmol/ml, normal range 6.07-27.1 nmol/ml). His progesterone (P) level was elevated (3.440 nmol/l, normal range 0.318-2.671 nmol/l). The diagnosis of hyposmia was confirmed by the olfactory test, showing the ability to distinguish alcohol, water, and vinegar, but not weak amyctic substances such as coffee and bread. The results of pituitary MRI showed that there was no abnormal signal, and the pituitary gland was slightly flattened. The patient was suspected of having KS, and therefore a GnRH stimulation test (100 μg GnRH, 2 ml physiological saline, intravenous injection) was performed. FSH and LH levels at 0, 30, 60, and 90 minutes after GnRH injection were tested and the levels of FSH were 0.860, 2.250, 2.950, 3.040 mIU/ml and the levels of LH were 0.400, 0.990, 1.020, 0.860 mIU/ml, respectively. The patient underwent whole-exome sequencing, which identified two genetic defects:

1) a missense mutation in the *KAL1* gene: a G to A transition at position1600 in exon 11, changing codon 534 encoding valine into isoleucine;
2) a missense mutation of *PROKR2* gene, a G to A transition at position 533 in exon2, changing codon 178 encoding tryptophane into serine (Fig. 2).

**Table 1:**
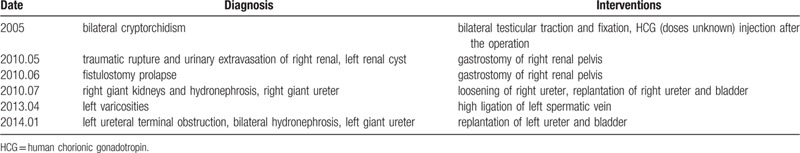
Surgical managements of KS and its complications.

Combined with the results of clinical manifestation, hormone test, GnRH stimulation test and whole-exome sequencing, the patient was diagnosed as KS. Idiopathic hypogonadotropic hypogonadism (IHH) and CHARGE syndrome, on the other hand, were also considered and precluded.

After 2 years of HCG and human menopausal gonadotropin (HMG) replacement therapy, his serum T reached normal level and Tanner stage II pubic hair development was observed. The details of hormone replacement therapy (HRT) and outcomes were showed in Table 2. However, the patient had no sperm detected in semen analysis.

**Table 2:**
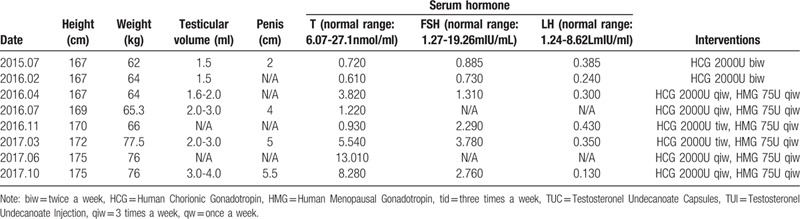
Hormone replacement therapy and outcomes.

## 3 Discussion

According to the literature, the incidence of KS ranges from 1/10,000 in males and 1/50,000 in females, which is 5 times higher in males than in females.^[4–6]^ The main causes of KS are the impairment of olfactory axis development and of GnRH neurons migration caused by gene mutations.^[6]^ The primary purpose of treatment for male patients is to promote masculine development, and the second is to restore the gonadal function and even fertility of patients who are eager to give birth.^[7]^

As one of the few treatable infertility, early diagnosis and treatment of KS are particularly important. Renal agenesis is the most common complication for KS patients, which may provide clues for the early diagnosis and treatment of the patient. Fourteen types of urinary disorders in the KS patients have been reported before, including renal agenesis (unilateral and bilateral),^[4]^ renal malrotation,^[8]^ bilateral dilatation of the calyces and pelves,^[8]^ horseshoe kidneys,^[9]^ multicystic dysplastic kidneys,^[10]^ vesiculourateral reflux,^[10,11]^ ectopic right ureteric orifice,^[12]^ left hydronephrosis,^[11]^ left reflux^[11]^ and hyderoureter.^[11]^ In this case, we reported 8 rare urinary disorders co-exist in one patient, in which bilateral giant kidneys, urinary extravasation of right renal, bilateral megalo-ureters, left ureteral terminal obstruction, bilateral renal cyst and bladder emptying disorder have not been reported before.

*KAL1* is a well-known gene most closely related to KS-associated renal agenesis. Moreover, renal agenesis was only found in male patients and occurred in 31.8% KS patients due to *KAL1* defects, consistent with the percentage of renal agenesis (31%) in X-linked KS patients in the study by Quinton et al.^[13]^ Therefore, we could imply that renal agenesis may be a marker of *KAL1* mutation in KS patients with an estimated sensitivity of 30%. In the present study, our patient was first detected with two-point mutations in the *KAL1* gene. However, one is a G-to-A transition at position 1600 in exon 11, changing codon 534 from valine to isoleucine with a carrier rate of 59.16%, suggesting that mutation at this position has no genotype-phenotype correlation. The other mutation is a synonymous mutation of a C-to-T transition in exon 12. Next, we performed a whole-exome sequencing for the patient and identified a G-to-A transition in exon 2 in the *PROKR2* gene, changing codon 178 from tryptophan to serine with a carrier frequency of 0.01813%. *PROKR2* is located on the autosomal chromosome region 20p13 and is mainly expressed in the brain, olfactory bulbs, and testes.^[14]^ To the best of our knowledge, KS patients with a *PROKR2* mutation presenting urinary disorders have not been reported elsewhere, and the associated genetic links were unclear. We suspected that a single mutation of KS-related genes may not have significant clinical performance, but multi-mutations may have an accumulation effect.^[2]^ Therefore, when co-mutation with other KS-related genes such as *PROKR2*, *KAL1* with p.V534I mutation may lead to a novel presentation of urinary disorders. We also searched for KS patients with urinary disorders in the absence of *KAL1* mutation. Remarkably, another *KAL1* mutation at position c.1600G > A (p. V534I) in exon11 in a patient with bilateral abnormal rotation and bimanual synkinesis was found.^[15]^ Whether this mutation interacts with other KS-related genes to cause certain clinical phenotypes in KS, or other genes associated with KS-related urinary disorders need to be explored is yet unclear. More cases are required for genome, transcriptome, proteomics, and metabolomics analysis.

In this report, the patient accepted bilateral testicular traction and fixation, and received HCG injection after the operation. As shown in Table 2, after two years of HCG and HMG treatment, the patient's T level reached normal level and developed male secondary sexual characteristics. However, the LH level of the patient was still lower than normal, and no sperm was detected in semen analysis. The results of the GnRH stimulation test also showed that the LH value was much lower than the normal value after GnRH stimulation. FSH and LH values were about 3 times higher than those before injection, indicating that the patient had normal GnRH response. GnRH treatment provides pulse management of GnRH via a mini-infusion pump, which should be used to induce spermatogenesis and fertility for our patient in future.^[16]^

## 4 Conclusions

In conclusion, we report 8 novel urinary disorders with missense mutations of *KAL1* and *PROKR2* in a case of KS for the first time, which enrich the integrity of urinary disorder types in patients with KS. Urinary disorders and cryptorchidism can be a hint for the occurrence of hypogonadotropic hypogonadism requiring the attention of doctors for early diagnosis and treatment. More studies on the genotype-phenotype correlation need to be done, especially between *KAL1* mutation and KS related urinary disorders. Identification of this correlation may be helpful for an early and precise diagnosis, and lays the foundation of timely and effective treatment.

## 5 Ethical approval

Institutional review board/ethics committee approval was obtained from the Institutional Review Board of the First Hospital of Jilin University.

## Acknowledgments

We want to acknowledge the patient and his family for their support and cooperation.

## Author contributions

**Conceptualization:** Huining Tian, Xiaokun Gang, Guixia Wang

**Data curation:** Huining Tian

**Formal analysis:** Huining Tian

**Funding acquisition:** Guixia Wang.

**Investigation:** You Lv, Lin Sun.

**Methodology:** Zi Yan.

**Project administration:** Guixia Wang, Xiaokun Gang.

**Resources:** Guixia Wang.

**Validation:** Guixia Wang, Xiaokun Gang.

**Visualization:** Guixia Wang, Xiaokun Gang.

**Writing – original draft:** Huining Tian.

**Writing – review & editing:** Zi Yan.
